# Supplementary material for: Causal Relationship between Aging and Anorexia Nervosa: A White-Matter-Microstructure-Mediated Mendelian Randomization Analysis
Source: Biomedicines. 2024 Aug 16;12(8):1874. doi: 10.3390/biomedicines12081874 (PMC11351342; doi:10.3390/biomedicines12081874)
Supplement: Supplementary file 1 [file biomedicines-12-01874-s001.zip › Supplementary Figure/Figure S4/Singe SNP plot of MR analysis for TL to ubm-b-1896.pdf]

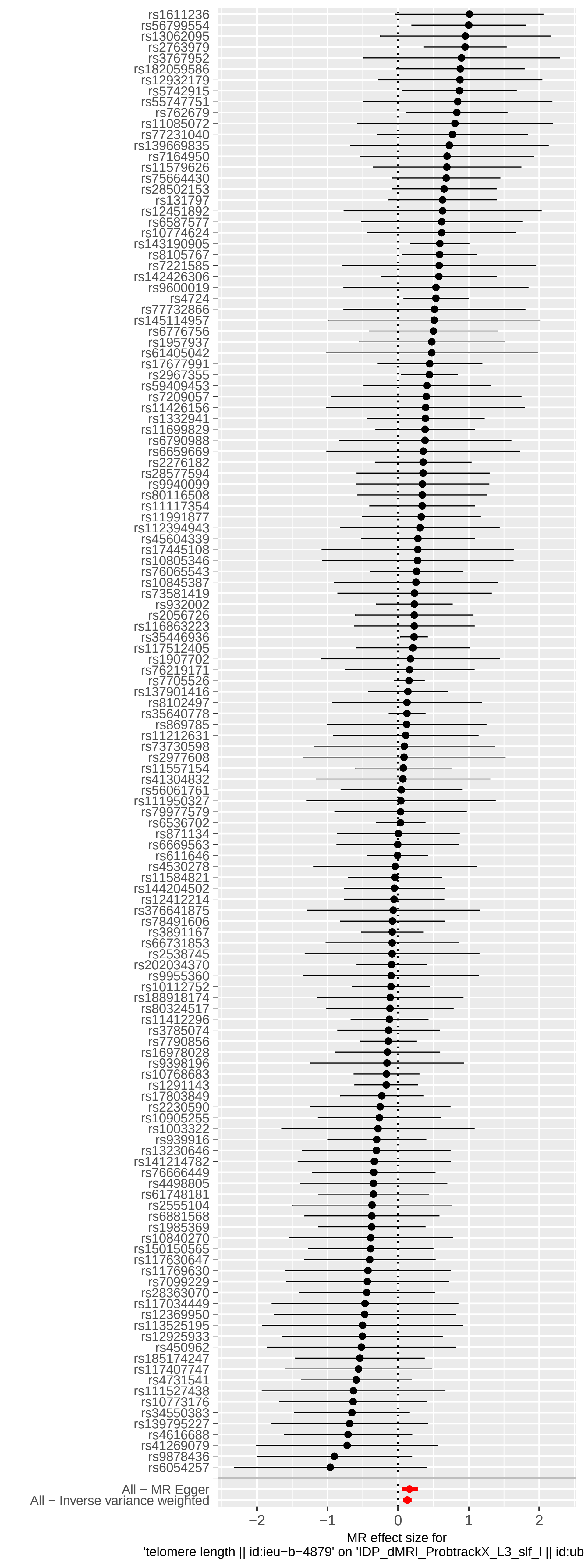
rs1611236
rs56799554
rs13062095
rs2763979
rs3767952
rs182059586
rs12932179
rs5742915
rs55747751
rs762679
rs11085072
rs77231040
rs139669835
rs7164950
rs11579626
rs75664430
rs28502153
rs131797
rs12451892
rs6587577
rs10774624
rs143190905
rs8105767
rs7221585
rs142426306
rs9600019
rs4724
rs77732866
rs145114957
rs6776756
rs1957937
rs61405042
rs17677991
rs2967355
rs59409453
rs7209057
rs11426156
rs1332941
rs11699829
rs6790988
rs6659669
rs2276182
rs28577594
rs9940099
rs80116508
rs11117354
rs11991877
rs112394943
rs45604339
rs17445108
rs10805346
rs76065543
rs10845387
rs73581419
rs932002
rs2056726
rs116863223
rs35446936
rs117512405
rs1907702
rs76219171
rs7705526
rs137901416
rs8102497
rs35640778
rs869785
rs11212631
rs73730598
rs2977608
rs11557154
rs41304832
rs56061761
rs111950327
rs79977579
rs6536702
rs871134
rs6669563
rs611646
rs4530278
rs11584821
rs144204502
rs12412214
rs376641875
rs78491606
rs3891167
rs66731853
rs2538745
rs202034370
rs9955360
rs10112752
rs188918174
rs80324517
rs11412296
rs3785074
rs7790856
rs16978028
rs9398196
rs10768683
rs1291143
rs17803849
rs2230590
rs10905255
rs1003322
rs939916
rs13230646
rs141214782
rs76666449
rs4498805
rs61748181
rs2555104
rs6881568
rs1985369
rs10840270
rs150150565
rs117630647
rs11769630
rs7099229
rs28363070
rs117034449
rs12369950
rs113525195
rs12925933
rs450962
rs185174247
rs117407747
rs4731541
rs111527438
rs10773176
rs34550383
rs139795227
rs4616688
rs41269079
rs9878436
rs6054257
All − MR Egger
All − Inverse variance weighted
−2
−1
0
1
2
MR effect size for
'telomere length || id:ieu−b−4879' on 'IDP_dMRI_ProbtrackX_L3_slf_l || id:ub
